# Cancer Risk Studies and Priority Areas for Cancer Risk Appraisal in Uganda

**DOI:** 10.5334/aogh.2873

**Published:** 2020-07-07

**Authors:** Alfred Jatho, Binh Thang Tran, Jansen Marcos Cambia, Miisa Nanyingi, Noleb Mugume Mugisha

**Affiliations:** 1 Department of Cancer Control and Population Health, National Cancer Center Graduate School of Cancer Science and Policy, Goyang, KR; 2 Uganda Cancer Institute, Kampala, UG; 3 Uganda Martyrs University, Kampala, UG; 4 Institute of Research and Development, Duy Tan University, Da Nang, VN

## Abstract

**Background:**

Research into aetiologies and prevention of the commonest cancers and implementation of primary and secondary prevention can reduce cancer risk and improve quality of life. Moreover, monitoring the prevalence of cancer risk factors in a specific population helps guide cancer prevention and early detection efforts and national cancer control programming.

**Objective:**

This article aims to provide the scope and findings of cancer risk studies conducted in Uganda to guide researchers, health-care professionals, and policymakers.

**Methods:**

Between November 2019 to January 2020, we searched peer-reviewed published articles in Pubmed, EMBASE and Cochrane Library (Cochrane central register of controlled trials-CENTRAL). We followed the recommendation of the Preferred Reporting Items for Systematic Reviews and Meta-Analyses – the PRISMA. The primary focus was to identify cancer risk and prevention studies conducted in Uganda and published in peer-reviewed journals from January 2000 and January 2020. We used key Boolean search terms with their associated database strings.

**Results:**

We identified 416 articles, screened 269 non-duplicate articles and obtained 77 full-text articles for review. Out of the 77 studies, we identified one (1%) randomized trial, two (2.5%) retrospective cohort studies and 14 (18%) case-control studies, 46 (60%) cross-sectional studies, five (6.4%) ecological studies, three panel studies (4%) and six (8%) qualitative studies. Cervical cancer was the most studied type of cancer in Uganda (23.4%, n = 18 studies), followed by lymphomas – both Hodgkin and Non-Hodgkin sub-types (20.7%), n = 16 studies) and breast cancer (15.6%, n = 12 studies). In lymphoma studies, Burkitt lymphoma was the most studied type of lymphoma (76%, n = 13 studies). The studies concentrated on specific cancer risk awareness, risk perceptions, attitudes, uptake of screening, uptake of human papillomavirus vaccination, the prevalence of some of the known cancer risk factors and obstacles to accessing screening services.

**Conclusion:**

The unmet need for comprehensive cancer risk and prevention studies is enormous in Uganda. Future studies need to comprehensively investigate the known and putative cancer risk factors and prioritize the application of the higher-hierarchy evidence-generating epidemiological studies to guide planning of the national cancer control program.

## Background

Cancer is the second leading cause of death worldwide, with over 18 million new cases and 9.6 million cancer deaths estimated to have occurred in 2018 [1]. By 2030, it is projected that there will be approximately 26 million new cancer cases and 17 million cancer deaths per year [1]. Approximately 50% of all new cancer cases and 70% of all deaths due to cancer worldwide occur in low- and middle-income countries and cancer burden in Africa is estimated to double by 2030 [2].

In Uganda, 32,000 new cases and 21,000 deaths caused by cancer occurred in 2018 and 56,238 people were living with cancer by 2018 [2]. According to the Globocan cancer statistics report of 2018 [2], the top seven cancers in Uganda – cancer of the cervix, KS, breast, prostate, NHL, liver and esophageal – account for 70% of new cancer cases. Late presentation that is estimated to stand at 80% and limited access to diagnosis and treatment services contribute to the high cancer death rate in Uganda.

The World Health Organization estimates that between 30–50% of all cancers are avoidable by preventing or reducing exposure to cancer risk factors.

Therefore, based on the current cancer incidence [1], a majority of the top seven cancers in Uganda, that account for 70% of new cancer cases, can be prevented by modifying their risk factors. Research into aetiologies of these most common cancers and implementation of primary and secondary prevention can reduce the risk of carcinogenesis and improve quality of life. Moreover, monitoring the prevalence of cancer risk factors in a specific population helps guide cancer prevention and early detection efforts and national cancer control programming [3].

### Objective

This review aimed to provide the scope of cancer risk studies conducted in Uganda and their findings to guide researchers and policymakers on the locally generated evidence and perspectives on current priority cancer risk appraisal.

## Method

Between November 2019 and January 2020, we searched peer-reviewed published articles in Pubmed, EMBASE and Cochrane library (Cochrane central register of controlled trials-CENTRAL), irrespective of years of publication. We followed the recommendation of the Preferred Reporting Items for Systematic Reviews and Meta-Analyses – the PRISMA. The primary focus was to identify cancer risk and prevention studies conducted in Uganda and published in peer-reviewed journals during January 2000 and January 2020.

### Study Identification and Selection Procedure

We used the following Boolean search terms with their associated database strings to identify literatures on cancer risk and prevention studies in Uganda: Uganda cancer risk, cancer risk factors, cancer case control studies, cancer cohort studies, cancer risk cross-sectional study, cancer epidemiology, neoplasm risk, tumour risk, tumorigenesis, carcinogens and carcinogenesis, and cancer prevention.

We further supplemented the search criteria to generate more published articles by using the ten most common types of cancer in Uganda and key risk factors in the search terms: breast cancer, cervical cancer, prostate cancer, Kaposi sarcoma, human herpes virus 8, liver cancer, esophageal cancer, lymphoma, leukaemia, blood cancer, stomach cancer, gastric cancer, helicobacter pylori, colon cancer, colorectal cancer, lung cancer, ovarian cancer, human papilloma virus, HIV cancer, hepatitis B virus, hepatitis C virus, Epstein bar virus, tobacco smoking, alcohol consumption, diet, nutrition and cancer, overweight, obesity and cancer, physical activity, exercise and cancer, and unhealthy lifestyles in Uganda.

Three cancer experts independently screened 416 titles and abstracts of the identified articles to evaluate their relevance to the study objective. A total of 269 non-duplicate articles were assessed for eligibility, of which 77 full-text articles that met the eligibility criteria were reviewed.

### Findings

We identified 416 articles, screened 269 non-duplicate articles and obtained 77 full-text articles for review (Figure 2). Out of the 77 articles, 71 were quantitative studies and six were qualitative studies that used narrative strategies of focus group discussions (FGDs) and key informant interviews (KIIs). The 77 eligible articles were published during January 2000 through January 2020; a period of 20 years.

### Classification of Studies by Epidemiological Designs

Out of the 77 studies, we identified one (1%) randomised trial, two (2.5%) retrospective cohort studies and 14(18%) case-control studies as the highest on the epidemiological ladder of evidence of original studies. The other studies were 46 (60%) cross-sectional studies, five (6.4%) ecological studies, three (4%) panel studies, and six (8%) qualitative studies.

### Scope of Studies by Cancer Sites

Out of the 77 studies on cancer risk and prevention conducted in Uganda, most (61%, n = 47) investigated cervical, lymphomas, and breast cancers. Cervical cancer was the most studied type of cancer in Uganda (23.4%, n = 18 studies), followed by lymphomas – both Hodgkin and non-Hodgkin sub-types (20.7%), n=16 studies) – and breast cancer (15.6%, n=12 studies). In the lymphoma studies, Burkitt lymphoma was the most studied type of lymphoma (76%, n = 13 studies).

The least studied types of cancer were Kaposi sarcoma (5.1%, n = 4 studies), liver cancer (5.1%, n = 4 studies), esophageal and gastrointestinal, excluding liver, cancer (3.8%, n = 3 studies), prostate cancer (2.6%, n = 2 studies), and conjunctival cancer (2.6%, n = 2 studies). The effect of HIV on cancer development and progression constituted 7.8% (n = 6 studies) of the studies.

Studies that examined the prevalence of cross-cutting risk factors of non-communicable diseases such as tobacco smoking, alcohol consumption, and dietary factors accounted for 6.5% (n = 5 studies). Three studies (4.0%) examined the trend in cancer incidence, one study assessed anogenital warts (1.3%, n = 1 study), and one study (1.3%, n = 1 study) developed breast and cervical cancer awareness tools. The scope of these studies is summarised in **Box 1**.

**Box 1: Scope of the types and aims of cancer risk studies conducted in Uganda from January 2000 to January 2020.**

**Table:** 

No	Type of cancer n (%)	Aims/scope of cancer risk and prevention studies done in Uganda from January 2000 to January 2020

1	Cervical 18 (23.4%)	These studies assessed awareness about cervical cancer risk factors, perceptions and attitudes, uptake of human papillomavirus (HPV) vaccination, sexual behaviour of the HPV-vaccinated and non-vaccinated young girls, perceived barriers to cervical screening, knowledge and attitudes of men about HPV, healthcare, patients’ factors and stage at diagnosis, self versus clinic-based collection of HPV specimens for cervical screening. Functional cervical health literacy, the intention of women to screen for cervical cancer, uptake and correlates of cervical screening among HIV-infected women, uptake of cervical cancer screening in rural communities, perceptions of community members on integration of cervical screening in HIV clinics, and acceptability of cervical screening integration into immunization clinics were also assessed.
2	Lymphomas 16 (20.7%)	These studies described the epidemiology of Epstein-Barr virus (EBV), prevalence of EBV, human herpes virus 8 (HHV-8), and human immunodeficiency virus (HIV)-1 in B-cell non-Hodgkin lymphoma, age-specific patterns of Burkitt lymphoma (BL) cases, malaria, and risk of endemic Burkitt lymphoma (eBL) and factors associated with time to diagnosis of BL cases. The next-generation sequencing (NGS) to detect B-cell receptor (BCR) gene rearrangements in eBL, oral human herpes virus shedding kinetics, EBV viral load, and serology were investigated.
3	Breast 12 (15.6%)	These studies investigated breastfeeding and breast cancer risk, impact of alcohol, effect of knowledge on prevention, perceived barriers to early detection, role of high serum estradiol, role of blood folate level, and risk of breast cancer by ER status. Breast self-examination practices, role of family obligation, and stress on women’s participation in preventive breast health services, efficacy of mass self-breast screening, relationship between benign breast tumour (BBD) and breast cancer, full-term pregnancy, and breast cancer risk were investigated.
4	Kaposi sarcoma 4 (5.1%)	These studies investigated the human herpes virus (HHV-8) DNA in plasma, characterized the HHV-8 transcriptome, the HHV-8 gene expression in KS tumors for identification of candidate biomarkers, and risk factors for HHV-8 DNA detection.
5	Esophageal and other gastrointestinal, excluding liver 3 (3.8%)	These studies determined the prevalence, trend, and distribution of gastrointestinal malignancies and estimated the population attributable fraction of smoking and alcohol to esophageal squamous cell carcinoma (ESCC) and characterized the burden of esophageal cancer.
6	Liver 4 (5.1%)	These studies focused on the prevalence of hepatitis B virus (HBV) infection, its risk factors and evaluated the prevention-behavioral intentions in regard to HBV and liver cancer.
7	Prostate 2 (2.6%)	These studies assessed the knowledge, attitudes, and practices of men regarding risk, prevention, and screening for prostate cancer.
8	Conjunctival 2 (2.6%)	Factors associated with conjunctival cancer, determining if conjunctival squamous cell carcinoma (CSCC) harbors human HPV DNA and if CSCC is associated with activation of epidermal growth factor receptor (EGFR) signaling pathway were investigated.
9	HIV and cancer 6 (7.8%)	These studies evaluated the association between anti-retroviral treatment (ART) and cancer incidence, how HIV infection influences the presentation and manifestation of cancer, HIV infection and stage of cancer at presentation for treatment. The role of HIV in cancer survival and well-being of cancer patients, frequency of genital HSV shedding in HIV-seropositive versus HIV-seronegative men and women were also evaluated.
10	NCDs-cancer related risk 5 (6.5%)	These studies described the prevalence of risk factors for non-communicable diseases (NCDS), including tobacco use and alcohol consumption in Uganda and assessed the willingness of tobacco farmers to stop growing tobacco.
11	Trend in cancer incidence 3 (4.0%)	These studies described the trends of the commonest cancers in Uganda using data from Kampala and Gulu population-based cancer registries.
12	Anogenital 1 (1.3%)	This study assessed the risk factors of anogenital warts.
13	Breast & cervical awareness tool 1 (1.3%)	This study developed and validated breast and cervical cancer awareness assessment tool.

## Findings of the Reviewed Studies

### Cervical cancer

Regarding cervical health (Table 1), awareness about risk factors among women is still low in Uganda, ranging from about 40% to 80% [4,5,6,7,8]. The uptake of cervical cancer screening is still low ranging from 7% in rural area and 30% in urban centres [9,10,11,12,13]. Moreover, intention to screen is very high, ranging between 60–90% [4,9].

**Table 1:** Summary of quantitative findings of cervical cancer risk studies conducted in Uganda from January 2000 to January 2020.

No	Authors, year	Study types	Sample size	Factor	Effect measure	Effect size (95% CI)	P-value

1	Mukama et al. 2017 [4]	Cross-sectional	900	Knowledge of at least one preventive measure of CC among women in Eastern Uganda	Proportion	62.4%	
2	Mwaka et al. 2015 [5,19]	Cross-sectional	448	Knowledge of CC risk factors among women in northern Uganda	Proportion	82.6%	
3	Mukama et al. 2017 [4]	Cross-sectional	900	Perceived risk of CC	Proportion	76%	
4	Mutyaba et al. 2006 [7]	Cross-sectional	300	Knew at least one risk factors of CC	Proportion	40%	
5	Mwaka et al. 2015 [5]	Cross-sectional	149	Financial difficulties and risk of late diagnosis	aOR	5.5 (1.58, 20.64)	
6	Mwaka et al. 2015 [5]	Cross-sectional	149	Late referral and risk of late diagnosis	aOR	13.0 (3.59–47.3)	
7	Mwaka et al. 2015 [5]	Cross-sectional	149	5–9 biological children and risk of late-diagnosis	aOR	0.27 (0.08– 0.96)	
8	Campos et al. 2017 [119]	Monte Carlo simulation model	–	HPV self-collection efficiency versus clinic sampling.	Minimum coverage for efficiency	75%	
9	Twinomujuni et al. 2015 [9]	Cross-sectional	416	Ever-screened for cervical cancer	Proportion	7%	
10	Twinomujuni et al. 2015 [9]	Cross-sectional	416	Intention to screen among those with sexual partner.	aPR	1.4 (1.11–1.68)	
11	Twinomujuni et al. 2015 [9]	Cross-sectional	416	Intention to screen among those unafraid of positive result	aPR	1.6 (1.36–1.93)	
12	Twinomujuni et al. 2015 [9]	Cross-sectional	416	Intention to screen among those with perceived high risk of CC	aPR	2.0 (1.60–2.58)	
13	Wanyenze et al. 2017 [10]	Cross-sectional	5198	Screening uptake among HIV-infected	% coverage	30.3%	
14	Wanyenze et al. 2017 [10]	Cross-sectional	5198	Lack of time for screening among HIV-infected	Proportion	25.5%	
15	Ndejjo et al. 2017 [4]	Cross-sectional	900	Intention to screen in general population	Proportion	91%	
16	Ndejjo et al. 2017 [4]	Cross-sectional	900	Willing to vaccinate their daughters against cervical cancer	Proportion	90.4%	
17	Ndejjo et al. 2016 [16]	Cross-sectional	900	Health worker’s advice as predictor for screening	aOR	87.85	0.001
18	Ndejjo et al. 2016 [16]	Cross-sectional	900	Knowing where screening services are offered as predictor for screening	aOR	6.24	0.004
19	Ndejjo et al. 2016 [16]	Cross-sectional	900	Knowing someone who had ever been screened as predictor for screening	aOR	9.48	0.001
20	Banura et al. 2008 [14]	Cross-over Case-control	987	Prevalence of HPV among women	PR	60%	
21	Kisakye et al. 2018 [15]	Cross-sectional	460	Uptake of HPV vaccination	% coverage	17.61%	
22	Kisakye et al. 2018 [15]	Cross-sectional	460	Effect of higher level of education on HPV vaccination uptake	aPR	1.48 (1.11–1.97)	
23	Kisakye et al. 2018 [15]	Cross-sectional	460	Effect of positive attitude on HPV vaccination uptake	aPR	3.46 (1.70–7.02)	
24	Kisakye et al. 2018 [15]	Cross-sectional	460	Effect of health worker’s advice on HPV vaccination uptake.	aPR	1.55 (1.15–2.11)	
25	Kisakye et al. 2018 [15]	Cross-sectional	460	Effect of Village Health Team on HPV vaccination uptake	aPR	3.47 (1.50–8.02)	
26	Kisakye et al. 2018 [15]	Cross-sectional	460	Effect of community outreaches on HPV vaccination uptake	aPR	1.47 (1.02–2.12)	
27	Kisakye et al. 2018 [15]	Cross-sectional	460	Effect of HPV vaccine availability on HPV vaccination uptake	aPR	4.84 (2.90–8.08)	
28	Moses et al. 2018 [120]	Cross-sectional	60	Men who have ever heard of HPV	% proportion	24.6%	
29	Wawer et al. 2018 [18]	Randomised trial	544 IG, 488 CG	Incidence of high-risk HPV infection is lower in women with circumcised sexual partners compared to uncircumcised.	IRR	0·77 (0·63–0·93)	0·008
20	Li et al. 2017 [121]	Cross-sectional	571	Effect of age on acceptability of cervical screening	OR	1.10	<0.001
31	Li et al. 2017 [121]	Cross-sectional	571	Effect of employment on acceptability of cervical screening	OR	2.00	0.019

* aPR = adjusted prevalence ratio, OR = Odds ratio, aOR = adjusted odds ratio, IRR = Incidence rate ratio, CC = cervical cancer, CI = confidence interval, IG = Intervention group, CG = Control group.

Prevalence of HPV among women is 60%, with the high-risk HPV16 at 8.4%, HPV18 at 5.8%, HPV51 at 8.7%, and HPV52 at 12.1% [14]. HPV-vaccination uptake in girls aged 10 years is still low, ranging from 17–23% [15], yet willingness of parents to vaccinate their daughters is high (90%) [16] and school-grade approach to HPV vaccination is more feasible than age eligibility [17]. In a randomised trial that enrolled 544 women in the intervention group and 488 women in the control group, the risk of high-risk HPV was significantly lower in women with circumcised sexual partners with incidence risk ratio of 0.77(0.63–0.93) compared to those with uncircumcised sexual partners [18]. Financial difficulties and limited screening facilities are obstacles to cervical cancer screening uptake [5,19]. Functional health literacy assessment on cervical cancer among women in Eastern Uganda found that the majority (96.8%) of the participants demonstrated limited level of functional cervical cancer literacy in five different domains with a mean score of 42% [20].

### Lymphomas

Among the lymphomas (Table 2), EBV viral is higher in BL compared to other NHL [21,22,23] and malaria is an important co-factor for endemic BL in Uganda [24,25,26]. Reactivity of eBL cases to severe malaria associated antigens (PfEMP1), Pf malaria SERA5 protein and group A CIDRα1·5 variant were significantly associated [27]. In a study on human herpes virus oral shedding kinetics, EBV shedding rate among HIV-positive mothers was higher than that of HIV-negative mothers [28]. However, median time of “total delay” to diagnosis of BL is still high, at 12.9 weeks (IQR 4.3–25.7) in Uganda [29].

**Table 2:** Summary of quantitative findings on lymphomas’ risk studies conducted in Uganda from January 2000 to January 2020.

No	Authors, Year	Study types	Sample size	Factor/variable	Effect measure	Effect size (95% CI)	P-value

1	Orem et al. 2014 [21]	Case- control	96 cases, 31controls	Whole-blood EBV viral load in BL compared to other NHL	OR	6.67 (1.32–33.69)	0.04
2	Orem et al. 2014 [21]	Case- control	96 cases, 31controls	Chronic inflammatory conditions and risk of NHL other than BL	OR	0.19 (0.07–0.51)	0.001
3	Tumwine et al. 2010 [22]	Cross-sectional	119	Prevalence of EBV in BL tumours	PR	92%	
4	Tumwine et al. 2010 [22]	Cross-sectional	119	Prevalence of EBV in diffuse large B cell lymphomas tumours	PR	34.8%	
5	Tumwine et al. 2010 [22]	Cross-sectional	119	Prevalence of HHV-8 in BL tumours	PR	0%	
6	Tumwine et al. 2010 [22]	Cross-sectional	119	Prevalence of HHV-8 in diffuse large B cell lymphomas tumours	PR	0%	
7	Gantt et al. 2016 [122]	Panel study	32	The 12-month incidence of postnatal infection with HHV-6B.	IR	76%	
8	Gantt et al. 2016 [122]	Panel study	32	The 12-month incidence of postnatal infection with CMV.	IR	59%	
9	Gantt et al. 2016 [122]	Panel study	32	The 12-month incidence of postnatal infection with EBV.	IR	47%	
10	Gantt et al. 2016 [122]	Panel study	32	The 12-month incidence of postnatal infection with, for HSV-1, and 0% for HHV-8.	IR	8%	
11	Gantt et al. 2016 [122]	Panel study	32	The 12-month incidence of postnatal infection with HHV-8.	IR	0%	
12	Gantt et al. 2016 [122]	Panel study	32	Association of maternal HIV-1 infection with EBV.	aHR	7.2 (2.4–22.2)	<.001
13	Gantt et al. 2016 [122]	Panel study	32	Association of breastfeeding with CMV.	aHR	5.0 (1.2–21.1)	0.03
14	Gantt et al. 2016 [122]	Panel study	49	Association of younger child contacts with CMV.	aHR	1.4 (1.0–2.0)	0.04
15	Derkach et al. 2019 [27]	Case-control	343 cases, 750 controls	eBL cases reactivity to severe malaria associated antigens (PfEMP1).	aOR	0.60 (0.41–0.88)	0.03
16	Derkach et al. 2019 [27]	Case-control	343 cases, 750 controls	eBL cases reactivity to Pf Malaria SERA5 protein.	X^2^trend		Ptrend 0.007
17	Derkach et al. 2019 [27]	Case-control	343 cases, 750 controls	eBL cases reactivity to group A CIDRα1.5 variant.	X^2^trend		Ptrend 0.034
18	Buckle et al. 2013 [29]	Cross-sectional	82	Median time of “total delay” to diagnosis of BL.	Median	12.9 weeks (IQR 4.3–25.7)	
19	Buckle et al. 2013 [29]	Cross-sectional	82	Median time of “guardian delay” from1st symptoms of BL to 1st health encounter.	Median	4.3 weeks (Range 0.7–149.9)	
20	Buckle et al. 2013 [29]	Cross-sectional	82	Median time of “health system delay” to 1st health encounter to BL diagnosis.	Median	2.6 weeks (range 0.1–16.0)	
21	Maziarz et al. 2017 [25]	Cross-sectional	1150	Pf malaria prevalence rate in northern Uganda.	Prevalence rate	54.8%	
22	Peprah et al. 2019 [24]	Case-control	862 cases, 2,934 controls	History of in-patient malaria treatment 12 months ago and risk of eBL	OR	2.55 (1.39, 4.67)	0.01
23	Peprah et al. 2019 [24]	Case-control	862 cases, 2,934 controls	Higher maternal income and risk of eBL	OR	0.27 (0.14–0.52)	P-trend 0.004
24	Peprah et al. 2019 [24]	Case-control	862 cases, 2,934 controls	Higher level of paternal education and risk of eBL	OR	0.59 (0.39–0.89)	P-trend 0.013
25	Peprah et al. 2019 [24]	Case-control	862 cases, 2,934 controls	Higher maternal education and risk of eBL	OR	0.51 (0.28–0.96)	P-trend 0.005

* aHR = adjusted hazard ratio, IC = incidence rate, PR = prevalence rate, aPR = adjusted prevalence ratio, aOR = adjusted odds ratio, CI = confidence interval, Ptrend = P-value for trend analysis, CMV = cytomegalovirus, EBV = Epstein Barr Virus, HHV = human herpes virus 1, 6, 8. Pf = Plasmodium falciparum, PfEMP1 = Plasmodium falciparum erythrocyte membrane protein-1, SERA5 = Serine repeat antigen 5, CIDRα1.5 = Cysteine-rich interdomain region-α1.5 protein, BL = Burkitt lymphoma, eBL = endemic Burkitt lymphoma, NHL = non-Hodgkin’s lymphoma.

### Breast cancer

The breast cancer related factors (Table 3) that were found of significant protective role were breastfeeding with OR 0.04(0.01–0.18) [30] and being parous, with increasing parity offering more protection [31]. The factors that significantly increased the risk of breast cancer include current alcohol consumption [32], obesity [33], history of benign breast disease compared to those without [34]. In a breast cancer genetic predisposition study in Uganda, patients were eleven-fold more likely to carry a mutation with a prevalence of 5.6% BRCA1, 5.6% BRCA2, 1.5% ATM, 1% PALB2, 0.5% CDH1, 0.5% TP53 and 0.5% BARD1 compared to controls (OR 11.34, 95% CI: 3.44–59.06; P < 0.001) [35]. Breast cancer awareness level in general population is still low [36], similarly knowledge and skills related to breast self-exam (BSE) practices among university students is low [37,38]. Community cancer awareness by health workers as a source of information and uptake of breast cancer prevention modalities were more significantly associated than other avenues such as radios and TVs (OR 4.03 [1.01–15.98]) [39].

**Table 3:** Summary of quantitative findings on breast cancer risk studies conducted in Uganda from January 2000 to January 2020.

No	Authors, Year	Study types	Sample size	Factor	Effect measure	Effect size (95% CI)	P-value

1	Galukande et al. 2016 [30]	Case-control	113 cases and 237	Effect of breastfeeding on the risk of breast cancer	aOR	0.04 (0.01–0.18)	
2	Qian et al. 2014 [32]	Case-control	2138 Cases & 2,589 controls	Current alcohol drinking and risk of breast cancer.	aOR	1.01 (0.55–1.85)	
3	Qian et al. 2014 [32]	Case-control	2138 Cases & 2,589 controls	Past alcohol drinking and risk of breast cancer	aOR	0.99 (0.57–1.75)	
4	Awio et al. 2012 [33]	Case-control	70 Cases & 70 controls	Relationship between level of serum estradiol and breast cancer risk in cases compared to controls			0.647
5	Awio et al. 2012 [33]	Case-control	70 Cases & 70 controls	Higher BMI index and risk of breast cancer	OR	1.02 (1.01–1.04)	
6	Awio et al. 2012 [33]	Case-control	70 Cases & 70 controls	Late onset of menarche and risk of breast cancer	OR	0.68 (0.52–0.90)	
7	Atuhairwe et al. 2018 [39]	Cross-sectional	400	Relationship between radio as source of information and uptake of breast cancer prevention modalities.	OR	1.94 (1.16–3.24)	
8	Atuhairwe et al. 2018 [39]	Cross-sectional	400	Relationship between TVs as source of information and uptake of breast cancer prevention modalities.	OR	1.82 (1.14–2.93)	
9	Atuhairwe et al. 2018 [39]	Cross-sectional	400	Relationship between community cancer awareness by health workers as source of information and uptake of breast cancer prevention modalities.	OR	4.03 (1.01–15.98)	
10	Atuhairwe et al. 2018 [39]	Cross-sectional	400	Relationship between knowledge of breast cancer risk and uptake of breast cancer prevention modalities.	OR	1.98 (1.20–3.27)	
11	Atuhairwe et al. 2018 [39]	Cross-sectional	400	Relationship between knowing symptoms of breast cancer and uptake of breast cancer prevention modalities	OR	3.09 (1.62–5.88)	
12	Galukande et al. 2013 [123]	Cross-sectional (Analytical)	113	ER negative tumors exhibited significantly higher-grade tumors			0.001
13	Katende et al. 2016 [37]	Cross-sectional	204	Level of breast cancer awareness among Makerere university students.	Proportion	98.0%	
14	Katende et al. 2016 [37]	Cross-sectional	204	Skills related to breast self-exam (BSE) practices among Makerere university students.	Proportion	43.6%	
15	Scheel et al. 2019 [40]	Cross-sectional	401	Effect of family obligation (FO) stress on women’s participation in preventive breast health awareness.	Regression PD	–0.02	0.008
16	Scheel et al. 2019 [40]	Cross-sectional	401	Effect of FO stress on women’s participation in breast health check-up.	Regression-PD	–0.02	0.018
17	Adedokun et al. 2019 [34]	Case-control	@(2405 cases and 2749 controls)	The risk of breast cancer among women with history of benign breast disease compared to those without	aOR	1.42 (1.13–1.79)	
18	Sighoko et al. 2015 [31]	Case-control	1995 cases and 2631 controls	Risk of breast cancer in a parous woman with her first FTP at 20 years relative to nulliparous	OR	0.76 (0.57–0.99)	
19	Sighoko et al. 2015 [31]	Case-control	1995 cases and 2631 controls	Risk of breast cancer in a parous woman with 1 pregnancy relative to nulliparous.	OR	0.69 (0.49–0.96)	
20	Sighoko et al. 2015 [31]	Case-control	1995 cases and 2631 controls	Risk of breast cancer in a parous woman with 2 to 5 pregnancies relative to nulliparous.	0R	0.66 (0.48–0.91)	
21	Sighoko et al. 2015 [31]	Case-control	1995 cases and 2631 controls	Risk of breast cancer in a parous woman with 6 or more pregnancies	OR	0.67 (0.47–0.94)	

* OR = Odds ratio, aOR = adjusted odds ratio, CI = confidence interval, PD = Probability difference per 1-point increase, aPR = adjusted prevalence ratio. @ = conducted in Uganda, Nigeria, and Cameroon.

However, family obligation (FO) stress impacted negatively on women’s participation in breast health awareness [40]. Extending early detection efforts in rural communities yield promising results to downstage breast cancer presentation (shifting late-staged breast cancer disease presentation to early-stage) to improve survival [41]. The technical challenge is that the standard breast cancer screening option of mammography was found to miss 27% of breast cancer disease that ultrasound was able to detect as proven with histological diagnosis [42].

### Other types of cancer and risk factors

Pertaining other risk factors (Table 4), the prevalence of daily tobacco use among adult Ugandans was found to be 9.2% [43] and men were more likely to be daily tobacco users with aOR of 5.51 [3.81–7.95] [43]. Hospitality places like bars, restaurants, and hotels are not protecting the public against exposure to tobacco smoke [44,45] coupled with the limited awareness of the harmful effect of tobacco smoke among the tobacco users [46]. Prevalence of alcohol consumption was 26.8% and high-end alcohol consumption accounted for 12.7% of overall alcohol consumption [47]. Daily consumption of five or more servings of fruits in rural Uganda is still low, at 7.2%, whilst consumption of five or more servings of vegetables is very low (1.2%) [48].

**Table 4:** Summary of quantitative findings on other cancer risk studies conducted in Uganda from January 2000 to January 2020.

No	Authors, Year	Study types	Sample size	Factor	Effect measure	Effect size (95% CI)	P-value

1	Kabwama et al. 2016 [43]	Cross-sectional	3983	Prevalence of daily tobacco use	Prevalence rate	9.2 %	
2	Kabwama et al. 2016 [43]	Cross-sectional	3983	Men are more likely to be daily tobacco users	aOR	5.51 [3.81–7.95]	
3	Kabwama et al. 2016 [47]	Cross-sectional	3,956	Prevalence of alcohol consumption	Prevalence rate	26.8%	
4	Kabwama et al. 2016 [47]	Cross-sectional	3,956	Prevalence of high-end alcohol consumption	Prevalence rate	12.7%	
5	Mondo et al. 2013 [48]	Cross-sectional	611	Physically active status in rural Uganda.		49%	
6	Mondo et al. 2013 [48]	Cross-sectional	611	Daily ate five or more servings of fruits in rural Uganda.	Prevalence rate	7.2%	
7	Mondo et al. 2013 [48]	Cross-sectional	611	Daily ate five or more servings of vegetables in rural Uganda.	Prevalence rate	1.2%	
8	Mondo et al. 2013 [48]	Cross-sectional	611	Obesity in men in rural Uganda.	Prevalence rate	4.9%	
9	Mondo et al. 2013 [48]	Cross-sectional	611	Obesity in women rural Uganda.	Prevalence rate	9.0%	
10	Shebi et al. 2013 [124]	Cross-sectional	1,080 KSHV+356 KSHV-	Plasma KSHV DNA in KSHV seropositivity persons.	Prevalence rate	95%	
11	Shebi et al. 2013 [124]	Cross-sectional	1,080 KSHV+356 KSHV-	Plasma KSHV DNA in KSHV seronegative persons.	Prevalence rate	5%	
12	Shebi et al. 2013 [124]	Cross-sectional	1,080 KSHV+356 KSHV-	KSHV DNA quantity in plasma was higher in male sex.	Prevalence rate		0.002
13	Shebi et al. 2013 [124,125]	Cross-sectional	1,080 KSHV+356 KSHV-	KSHV DNA quantity in plasma was higher in rural compared to urban.	Prevalence rate		0.002
14	Rose et al. 2018 [126]	Cross-sectional	22 KS biopsies	KS tumors with a latent phenotype had high levels of total KSHV transcription than tumors with a lytic phenotype			
15	Rose et al. 2018 [126]	Cross-sectional	22 KS biopsies	Morphologically distinct KS tumors from the same individual exhibited similar KSHV gene expression profile.			
16	Phipps et al. 2015 [127]	Cross-sectional	48 KS biopsies	KS tumors expressed high levels of both latent and lytic HHV-8 mRNA transcripts.			
17	Phipps et al. 2015 [127]	Cross-sectional	48 KS biopsies	Genes encoding cytokines (vIL-6), growth regulatory genes (v-CYC), and apoptosis inhibitors (v-FLIP) were associated with different tumor types.			
18	Nalwoga et al. 2019 [125]	Cross-sectional	878	Detectable KSHV in blood decreases with age	Prevalence rate	22–23%	
19	Nalwoga et al. 2019 [125]	Cross-sectional	878	Detectable KSHV in saliva increases with age up to 12 years and subsequently decreases with increasing age	Prevalence rate	30–45%	
20	Nalwoga et al. 2019 [125]	Cross-sectional	878	More males (29%) than females (19%) shed KSHV DNA in saliva.	Prevalence rate		0.008
21	Nalwoga et al. 2019 [125]	Cross-sectional	878	Individuals with a current malaria showed higher levels of KSHV DNA in blood	Prevalence rate		0.031
22	Ocama et al. 2008 [53]	Cross-sectional	216	Esophageal squamous cell carcinoma is most prevalent in Uganda	Prevalence rate	98%	
23	Ocama et al. 2008 [53]	Cross-sectional	216	Esophageal cancer of upper third is of squamous cell type	Prevalence rate	100%	
24	Obayo et al. 2017 [52]	Ecologica	1468	The esophageal cancer is commonest gastro-intestinal malignancies over a 10-year period.	Prevalence rate	28.8% of the GIM	
25	Obayo et al. 2017 [52]	Ecologica	1468	The distribution of gastro-intestinal malignancies differs by regions.	Prevalence rate		0.001
26	Okello et al. 2016 [54]	Case-control	67 cases and 142 controls	PAF of ESCC due to smoking.	PAF	16	
26	Okello et al. 2016 [54]	Case-control	67 cases and 142 controls	PAF ESCC due to alcohol.	PAF	10	
27	Okello et al. 2016 [54]	Case-control	67 cases and 142 controls	Combined PAF of ESCC due to smoking and alcohol.	PAF	13%	
28	Bwogi et al. 2009 [55]	Cross-sectional	5875	National prevalence of hepatitis B virus (HBV) infection by HBsAg test.	Prevalence rate	10.3% (9.5–11.1)	
29	Bwogi et al. 2009 [55]	Cross-sectional	5875	Prevalence of HBV infection is highest in North-Eastern Uganda.	Prevalence rate	23.9%	< 0.001
30	Bwogi et al. 2009 [55]	Cross-sectional	5875	Prevalence of HBV infection in Northern Uganda is the second highest.	Prevalence rate	20%	< 0.001
31	Nankya-Mutyoba et al. 2019 [128]	Cross-sectional	455	Perceived risk and intention to screen for HBV was inversely associated.	PRR	0.95(0.90–1.00)	0.055
32	Nankya-Mutyoba et al. 2019 [128]	Cross-sectional	455	Perceived self-efficacy was positively associated with intention to screen for HBV.	PRR	1.18(1.10–1.23)	0.005
33	Kang et al. 2015 [59]	Longitudinal evaluation	713	Prevalence of aflatoxin in human serum	Prevalence	90%	
34	Du Z et al. 2018 [50]	Case-control	571 cases and 485 controls	In GWAS, the 8q24 risk region including rs72725854 was found a major contributor to Pca risk in Ugandan men	OR	3.37	P = 2.14 × 10–11
35	Du Z et al. 2018 [50]	Case-control	571 cases and 485 controls	Proportion of Pca risk accounted for by the African ancestry-specific risk variant rs72725854.	Proportion	12%	
36	Nakandi et al. 2013 [49]	Cross-sectional	545	Perceived susceptibility to Pca risk	Proportion	63.5%	
37	Nakandi et al. 2013 [49]	Cross-sectional	545	Intention to screen for Pca	Proportion	22.9%	
38	Nakandi et al. 2013 [49]	Cross-sectional	545	Knowledge on Pca risk	Proportion	10.3%	
39	Newton et al. 2002 [129]	Case-control	60 cases and 1214 controls	Conjunctival cancer was positively associated with HIV infection	OR	10(5.2–19.4)	<0.001
40	Yu et al 2010 [130]	Cross-sectional	38	Prevalence of HPV-18 genotype in conjunctival tumours	Prevalence rate	61%	
41	Yu et al 2010 [130]	Cross-sectional	38	Prevalence of HPV-16 genotype in conjunctival tumours	Prevalence rate	16%	
42	Yu et al 2010 [130]	Cross-sectional	38	Relationship between cytoplasmic p-MAPK and conjunctival tumor invasiveness.			0.05 or
43	Yu et al 2010 [130]	Cross-sectional	38	Relationship between cytoplasmic p-Akt and conjunctival tumor invasiveness.			0.028
44	Yu et al 2010 [130]	Cross-sectional	38	Relationship between EGFR signaling pathway expression and conjunctival tumor invasiveness			0.01
45	Mutyaba et al. 2015 [131]	Ecological	12,263	Availability of ART decreased the incidence of KS.	Proportion	5%	
46	Mutyaba et al. 2015 [131]	Ecological	12,263	Availability of ART decreased the incidence of stomach cancer.	Proportion	13%	
47	Menon et al. 2017 [132]	Case -control	449 cases and 282 controls	HIV-positive patients were less likely to present for care at an advanced stage.	OR	0.53(0.30 to 0.94)	

* OR = odds ratio, aOR = adjusted odds ratio, CI = confidence interval, KSHV = Kaposi’s sarcoma associated herpesvirus, PR = prevalence rate, aPR = adjusted prevalence ratio, GIM = gastro-intestinal malignancies. PAF = population attributable fraction, Pca = prostate cancer, ART = anti-retroviral therapy, + = Positive, – = Negative.

The level of prostate cancer awareness and intention to screen among Ugandan men is low [49], and genetic predisposition was observed in genome-wide association study (GWAS) to contribute significantly to the risk of developing prostate cancer among Ugandan men [50,51].

In gastrointestinal cancers, esophageal cancer is the commonest gastrointestinal malignancies (GIM), accounting for 28.8% [52]. Esophageal squamous cell carcinoma (ESCC) is most prevalent (98%) phenotype of esophageal cancer in Uganda [53]. PAF of ESCC due to smoking and alcohol are 16% and 10% respectively [54]. The national prevalence of hepatitis B virus (HBV) infection by HBsAg test was found at 10.3% (9.5–11.1), with the highest prevalence (23.9%) in northeastern Uganda [55].

Persons infected with HIV or syphilis are significantly more associated with prevalent HBV infection [56]. One in eight pregnant women (12%) are HBV positive [57] while health workers are at risk of occupational exposure to HBV [58]. Prevalence of HBV and its associated risk among health workers were 8.1% seroprevalence of current HBV, 48.1% prevalence of lifetime exposure to HBV infection, 67.8% of needle stick injuries, and 41.0% exposure to mucous membranes [58].

Exposure to aflatoxins (AF) based on archived serum from human immunodeficiency virus (HIV)-seronegative participants in south-western Uganda is very high (90%) [59] and the generalised-estimating equations indicated significant differences between the AFB_1_–lysine (AFB-Lys) adduct levels and agricultural occupations (*p* = 0.02) and rural residence (*p* = 0.05) [59].

### Trends in cancer incidence

The twenty-year trends in cancer incidence in Uganda from 1991–2010 from Kampala cancer registry, the longest series of cancer incidence surveillance in Africa since 1954, have shown an annual increase in incidence by 1.8% in cervical uteri and 3.7% in breast cancers [60]. This annual increase in cases of breast cancer was about double that of cervical cancer at 3.7% per annum.

In both cervical and breast cancers, the annual increase in incidences were more in the older age group than the younger age group – 5.2 % compared to 1.3 %, respectively [60]. The annual incidence of esophageal cancer has remained relatively constant over the 20-year period, with no significant difference since 1960 [60,61]. This could mean that exposure to the known and not well-known risk factors are entangled in our relatively societal inelastic environmental, lifestyles, and livelihood conditions, among others.

In Northern region of Uganda [62], the top three most common cancers in women were cervix (57/100,00 women), breast (12.7/100,000), and non-Hodgkin lymphoma (10.1/100,000) while in men it was prostate (20.4/100,000) and liver (12.8/100,000) and Kaposi sarcoma (11/100,000) were the most common. On Burkitt lymphoma, Ogwang et al. [63] found that the age-standardized incidence of Burkitt lymphoma was 2.4 per 100,000 people and was highest in 5–9-year-old age group with 4.1 per 100,000 people. The incidence was observed to be lower in districts far from the main hospital in Northern Uganda – St Mary Lacor hospital.

### Qualitative findings

The main individual-level barriers to primary and secondary prevention of cancer included inadequate level of cancer knowledge, attitude, and beliefs [4,12,64], fear of positive screening results, and apathy [65]. Regarding integration of cervical screening in HIV and immunisation clinics, worries that integration would increase waiting time for services at the health facility [66], fears of being detected positive for both cervical cancer and HIV [66], and financial constraints [4] were reported.

On availability of services, privacy, and comfort: lack of awareness regarding available cancer preventive services, exposure of women’s private body parts, perceived pain during screening, and men’s lack of support to women [67] were reported.

In the health system and policy arena, important health services issues that need urgent attention include the burdens of competing health care priorities [65], lack of the required basic cancer knowledge, and lack of skills among health workers in both private and public health facilities [5,67,68] to help their clients.

## Discussion and Perspectives

### Summary of findings

In this review, we found that the most studied types of cancer were cervical, lymphoma, especially Burkitt lymphoma and breast cancer. Interaction of HIV and cancer came fourth among the most cancer risk studies conducted in Uganda. Other types of cancers, for example, esophageal and liver cancer are less studied yet they exhibit the worst prognosis and lack programmatic screening options. Esophageal cancer is the third while liver cancer is the fifth cause of cancer mortality in Uganda. Research in the aetiologies and primary prevention of cancers like esophageal and liver cancer be could be the best life-saving option.

Cervical, breast, and prostate cancer screening is very low in Uganda. For example, cervical health screening coverage ranges from 7% in rural areas to 30% in urban centres [9,10,11,12,13]. The relationship between community cancer awareness by health workers as a source of information and uptake of breast cancer prevention modalities was more significantly associated than other avenues such as radios and TVs [39]. Therefore, if the district primary health care workers are equipped with the right information on primary prevention and early detection of cancer, the level of community awareness on cancer and engagement in preventive health behaviours could improve significantly. Moreover, for example, intention-to-screen for cancers is very high, ranging between 60–90% [4,9].

With the high (60%) prevalence of HPV among Ugandan women, more so, the high-risk HPV16 at 8.4%, HPV18 at 5.8%, HPV51 at 8.7 and HPV52 at 12.1% [14] amidst low HPV-vaccination uptake in girls aged 10 years is low (ranging from 17–23% [15], concerted efforts in risk reduction research including health behavioural intervention trials and primary prevention is required. This effort can leverage from the current high (90%) willingness of parents to vaccinate their daughters is high [16] and the feasibility of a school-grade approach to HPV vaccination [17].

Pertaining to the lymphomas, EBV is the main risk factor of BL compared to other NHL [21,22,23], while malaria infection is an important co-factor for endemic BL in Uganda [24,25,26]. In breast cancer risk studies, breastfeeding [30] and being parous, with increasing parity, offers more protection [31]. Therefore, encouraging mothers to breastfeed their babies as recommended in the child health program could be beneficial to women. However, current alcohol consumption [32], obesity [33], history of benign breast disease compared to those without [34], and genetic predisposition [35] were found to increase the risk of breast cancer in Uganda. Population-based operational research on how to engage individuals and communities to reduce their exposure to such risk factors remain important areas for research agenda.

In the qualitative assessment, we found that the main individual-level barriers to primary and secondary prevention of cancer in Uganda were: inadequate level of cancer awareness, negative attitude and beliefs, fear of positive screening test results, and apathy. However, family obligation (FO) stress reduced the capacity of women to participate in preventive health activities [40]. Therefore, extending primary prevention early detection of cancer services in rural communities could downstage presentation from late-staged to early-stage cancer to improve survival [41].

The health system and policy issues affecting access to primary and secondary prevention of cancer in Uganda were the burden of competing health care priorities, lack of the required basic cancer knowledge and skills among the primary health care workers, and limited cancer screening facilities. The technical challenge that the standard test for breast cancer screening option of mammography misses 27% [42] of breast cancer disease that ultrasound could detect needs urgent breast screening policy review to adopt or add the use of portable ultrasound scan to improve breast cancer screening validity.

It is crucial to note that many studies in African Countries, especially in Uganda remain shelved in universities’ and hospital libraries due to many factors, including lack of article publication fees, limited skills in writing a manuscript that meet publication standards required by the journals. The good news is that some organisations and journals have come up to offer research and publication mentorship, waiver of article processing fees, and access to free online databases for researchers in low-income countries [69]. However, we do not know if many of the Ugandan researchers are aware of these opportunities.

### Perspectives on current priority for cancer risk appraisal in Uganda

Based on what the previous studies investigated in Uganda, we recommended and discussed in the following priority research areas as our current perspectives of cancer risk appraisal needs in Uganda.

### Research on etiology of the leading cause of cancer mortality in Uganda

**Figure 1:**
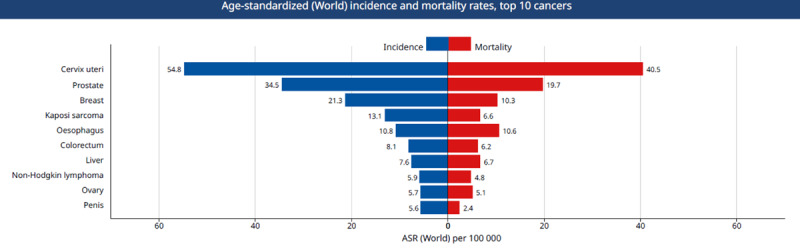
Top 10 causes of cancer mortality in Uganda. Source: Globocan 2018, IARC.

Comprehensive investigation into the known and putative risk factors of the leading cause of cancer mortality in Uganda is needed. The studies conducted so far did not comprehensively consider the known and putative risk factors linked or suspected to be linked to the type of cancer investigated. Such etiological studies should prioritise the top 5 if not the top 10 leading causes of cancer mortality in Uganda, especially those with the worst prognosis. For example, the top leading causes of cancer mortality are cervical, prostate, esophageal, breast, and liver cancers with age-standardized mortality rate of 40.5/100,000 women, 19.7/100,000 men, 10.6/100,000 persons, 10.3/100,000 persons, and 6.7/100,000 persons respectively (Figure 1).

**Figure 2:**
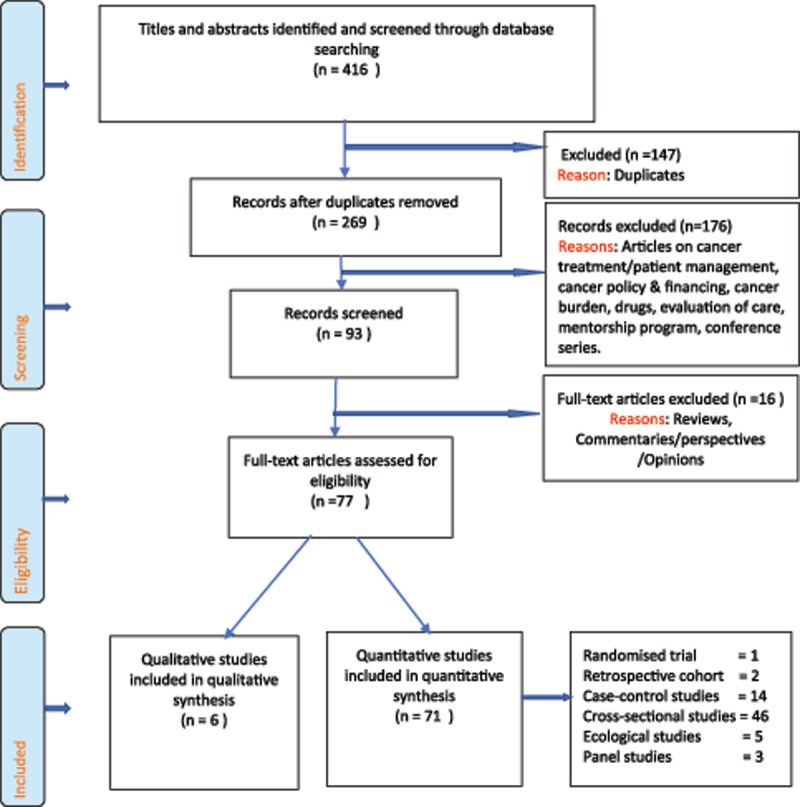
PRISMA Flow chart of cancer risk and prevention studies.

Of the top five causes of cancer mortality in Uganda, esophageal and liver cancers are characterized by very poor prognosis with an annual incidence of 10.8/100,000 persons versus mortality of 10.6/100,000 persons for EC and annual incidence of 7.6/100,000 persons versus mortality 6.7/100,000 persons for liver cancer. Comprehensive identification of important risk factors, including sociocultural variables that underpin health behavior is essential for effective prevention and evaluation of cancer control program, especially in low-resource settings.

### A comprehensive study on population attributable fractions of the known and putative cancer risk factors in Uganda

The contribution of a known risk factor to a specific cancer disease or a death is estimated by the population attributable fraction (PAF), also termed as population attributable risk (PAR). PAR is a public health measure of the proportion of a disease in the population due to exposure to a specific risk factor that could be avoided if the exposure or the risk factor was eliminated under an ideal exposure scenario consideration [70]. In the reviewed studies, only one study by Okello et al. [54] investigated attribution of two risk factors, cigarette smoking and alcohol consumption to esophageal cancer, in which both smoking and alcohol contributed a fraction of 13%, the other 87% are due to other putative factors that were not investigated. Population attributable risk (PAR) is used in quantification of the burden of disease and associated modifiable risk in a population [70]. Knowledge of population attributable risk or fraction (PAR) of modifiable cancer risk factors is important in prioritizing health promotion and specifically cancer prevention interventions. PAR is also invaluable for evaluation of cancer primary prevention and control efforts [71] and guides cancer control policies [72,73]. It is crucial to note that population attributable risk (PAR) estimates are dependent on specific risk factor prevalence, which is variant over time and are population group-specific [74,75], thus population specific assessment is a prerequisite. Therefore, PAR is an important tool for negotiating with policymakers of the benefits of cancer prevention interventions and informing them about likely costs of inaction to the population health. It is also useful in prioritizing the program interventions that are likely to yield the greatest public health impact and the return on investment – the best-buys scenario.

### Monitoring the population cancer risk trends

Monitoring the prevalence of cancer risk factors in a specific population helps guide cancer prevention and early detection efforts [3]. Emphasis should be put on prevalence of risk factors that are known to be associated with the top ten causes of cancer mortality in Uganda (Figure 1). Therefore, investment in surveillance of cancer prevention and early detection metrics is needed to generate evidence for population specific and national cancer control planning. Collection of baseline and outcome data for cancer prevention and control programs are necessary for evaluation and forecasting future funding and policy review. Development of comprehensive national cancer control plans is dependent on availability of such monitoring data, knowing that resources tend to be insufficient in cancer control programs; therefore, allocative efficiency is needed based on the trends of the most important risk factors.

### Analysis of age-period-cohort (APC) effects using methods that address identification problem (ID) of APC

The age-period-cohort (APC) effects are the changes in the patterns of incidence or mortality rates of a specific disease or condition in a specified population due to independent effects of age groups, calendar periods of diagnosis, and birth cohorts [76]. In cancer epidemiology, the APC effect framework includes parameters that describe the independent relationships between the rate of specific type of cancer and attained age, calendar period (year of cancer diagnosis), and birth cohort (year of birth). Generally, APC analysis helps us describe the complex historical, social, biological, and environmental etiological factors that simultaneously impact individual and population health [77,78,79]. This is important in explaining the suspected biological and social determinants of health.

There are now alternative statistical methods of addressing the identification problem (ID) of APC analysis, that is, the failure of the statistical models like regression models to estimate the independent effect of age, birth cohort, or period to the observed disease incidence or mortality. To address the above limitation, Yang, Land, and Fu suggested APC analysis using the intrinsic estimator (IE) for age-period-cohort analysis [76,80] and the cross-classified random effect model (CCREM) that apply a multi-level analytic framework and the hierarchical APC (HAPC)-growth curve model (GCM) [81].

The hierarchical age-period-cohort-growth curve model using accelerated longitudinal panel data (HAPCGCM-ALPD) can identify intra-cohort and inter-cohort variations in health status with age, explain health inequalities throughout the life cycle which other models cannot predict [76]. According to Heo et al. [76], there are three most useful data that can be analyzed for APC effects. Tabular age by period data can be analyzed well using the intrinsic estimator (IE)-APC models. Repeated cross-sectional data can be analyzed well by the hierarchical cross-classified random effects models (HAPC-CCREMs) while it is better to analyze the accelerated longitudinal panel data using the hierarchical APC-growth curve models [76].

### Health behavioral intervention trials and models in the context of cancer risk reduction

A recent study indicated limited level of functional cervical cancer health literacy among women in Eastern Uganda [20], therefore, implementation research into how to improve functional, communicative, and critical health literacy in the context of cancer prevention is needed. Population-based participatory research, especially how to use low-cost technology options for cancer screening and behavioral modification interventions for cancer risk reduction is needed. Also, relationship between knowledge, attitudes, beliefs, and cancer preventive health behaviors are crucial areas for both quantitative and qualitative research. Scholars have also reported that culturally mediated factors influence capacity of individuals and social groups to take control over determinants of their health [82]. Careful assessment of these factors could elicit them to the surface where programme implementers and policy makers can have a glance of them to guide health programme decision.

The applicability of health behavioral models and theories that explain and predict health behavior change at intra-personal, interpersonal, community-wide, organisational, and policy levels need to be tested in Ugandan populations. It is also important to investigate the cultural adaptations of the existing behaviour change theoretical models and how to provide and stimulate adoption of cancer preventive interventions, especially in rural areas that lack or have limited facilities and expertise for cancer prevention and early detection.

Many health promotion models and theories exist for use in influencing behavior change at individual, inter-personal, community, and organizational or policy level. At individual level, the rational model (RM), the health belief model (HBM), the extended parallel process model (EPPM), the transtheoretical model (TTM)/stages of change model (SCM), the activated health education model (AHEM), the precaution adoption process model (PAPM), motivational interviewing and brief interventions (MIBI), the elaboration likelihood model (ELM) of persuasion, the theory of planned behavior (TPB), and stimulus response theory (SRT) are the widely used models and theories with specific contextual applications and limitations [83,84,85,86,87].

At interpersonal level, social cognitive theory (SCT) and Social support/networks (SS/N) are common while at community wide level, communication theory (CT), diffusion of innovation (DOI) theory, community organizing/Rothman’s framework and PEN-3 cultural model. At the organizational and policy level, agenda-setting theory (AST), Milio’s framework for healthy public policy and the four-stage model for organizational change have been proposed and but have application challenges [88,89]. This is crucial because socio-contextual factors at various strata, individual, interpersonal, local community, health system organization, and international level influence decisions and health behaviors [90,91,92]. Clear description of intervention models, mode of application, measurement of constructs, concepts’ measuring tools, time-to-follow-up, outcome assessment, adoption, and sustainability of the changes need to be provided [93]. Furthermore, translation of cancer control evidence must be within the local context, otherwise the benefits of the known novel interventions tested in other settings may not be realized [94]. Cancer risk factor reduction interventions should be part of the priority areas of the mainstream cancer care model [95] and integrated in all levels of health service delivery and other NCD programs and other societal sectors.

### Health communication, interaction between the mass media and cancer control efforts in Uganda

Mass media such as TVs, radios, newspapers, and social media controls the biggest portion of how and which health information reaches the public. Media also plays a pivotal role in influencing health policy within their media coverage [96]. The mass media content in Uganda also includes media from alternative health practitioners such as herbalists and spiritual healers, among others that tend to enjoy the biggest coverage due to their ability to pay for mass media airtime driven by profit maximisation motive. This requires strategic health communication with appropriate engagement of the mass media fraternity [97,98]. However, exaggeration, underestimation, or misrepresentation of cancer health information can have profound consequences on public health.

Operational research into effective ways of delivering cancer health information, culturally sensitivity, attaining competitive equilibrium relative to the alternative competitors in the health sector such as the herbalists and cost-effective ways of benefiting from mass media should be prioritized to ensure that cancer prevention messages are accurate and reflects what is currently known and what is not known, what can and cannot be prevented, what can be cured or managed and what cannot be cured, as well as where to obtain such help.

### A genome-wide association study (GWAS)

GWAS is an approach used in genetics research to associate specific genetic variants with particular disease(s) [99,100]. This method searches the genome for small variations, called single nucleotide polymorphisms (SNPs), that occur more frequently in people with a particular disease than in people without the disease [101]. Once the new genetic associations are identified, researchers can use the information to develop better strategies to prevent, detect and treat the disease [102]. GWAS is deepening understanding of the genetic origins of many cancers that were not known or vaguely described. Health professionals will be able to use such tools to provide clients with individualized information about their risks of developing certain types of cancer [103]. The information will enable health professionals to tailor cancer prevention interventions to each person’s unique genetic makeup. If a person develops cancer, the information can be used to select the treatments most likely to be effective and least likely to cause adverse reactions in that particular patient. Therefore, GWAS is facilitating the development of “personalized cancer management” in the care of the individual as opposed to the current “one-size-fits-all” approach to cancer care. Although access to certain human tissues is challenging, in vitro differentiation of human induced pluripotent stem cells (iPS), which can be differentiated into cell types, offers the potential for disease-associated variants to be investigated [104].

### Molecular pathological epidemiology (MPE): Era of big-data health science and precision oncology

MPE is an integrative field that utilizes molecular pathology to incorporate interpersonal heterogeneity of a disease process into epidemiology as core field in era of big-data health science and precision medicine as opposed to the traditional epidemiology [105,106]. Traditional or conventional epidemiology assumes that individuals with the same disease entity have similar causes, show similar natural history of the disease, and experience similar responses to treatment or intervention [105,106]. This an assumption of “homogeneity” or “generalizability premise [107].”

MPE is based on “the unique disease principle” and “the disease continuum theory”. The disease continuum theory [105,106] states that “people diagnosed with different diseases can have overlapping aetiologies and pathogenesis” while the unique disease principle [107] states that “while people diagnosed with the same disease entity share some similarities, each individual has a unique pathologic process”. This is because each disease process results from unique profiles of exposomes, epigenomes, transcriptomes, proteomes, metabolomes, microbiomes, and interactomes with the macro-environment and tissue micro-environment [108,109].

In oncological context, exposomics deals with the assessment of an individual lifetime’s exposures to known cancer risk factors and how those exposures such as environmental factors, lifestyle factors like cigarette smoking, alcohol consumption, dietary patterns, among others interact with physiology, genetics, and epigenetics to dictate health status outcome. Exposomics involves application of both internal and external exposure assessment techniques. Internal exposure risk assessment includes genomics, lipidomics, transcriptomics, and proteomics [109], while external exposure assessment deals with environmental, occupational, and lifestyle-related factors.

Application of MPE therefore, addresses the need to investigate the inherent heterogeneity of pathogenic processes even for a single disease entity because in each individual, the development and progression of a disease are determined by a unique combination of exogenous and endogenous factors [105,106,110,111], thus resulting in different molecular and pathological subtypes of the disease. In addition to molecular features, host immune status and microbiome profile are likely to affect a disease process, and thus serve as informative biomarkers [112,113,114].

Evidence from MPE nosology can further provide a specific risk estimate for each disease subgroup, thereby enhancing the impact of genome-wide association studies on public health. MPE enables the exploration of whether an exposure forms a differential relationship with disease subgroups classified by molecular biomarkers [115], thus strengthening evidence for causal relationships.

Therefore, MPE demonstrates the relationship between an exposure and specific molecular alterations, refines the effect size of the association between an exposure and a specific disease subtype, supports causality, and uncovers the risk factors for a specific disease subtype that could be masked without subtyping the cancer tumor [116]. MPE can also be used to identify disease subtypes associated with benefits from lifestyle or pharmacological intervention and discover and validate molecular biomarkers for risk appraisal, early detection, diagnosis, and decision making on interventions.

The global challenges of inadequate tissue specimens sample size and paucity of interdisciplinary experts in MPE, especially in low-income countries such as Uganda and other African countries, can be overcome through international data sharing and world-wide collaborative consortia [116]. This could help to collect large-scale data from different parts of the world to increase the statistical power and generalizability of study findings [117,118]. Given the increasing availability of omics data on host and tumour when combined with environmental, behavioral, microbial, and immune profiles, this new MPE nosology could further promote the local and global trend of precision oncology.

### Strengths and Limitations of this Review

This study provides an insight in to the types of cancer whose risk factors have been investigated and those that have never been investigated among the Ugandan population. This could guide cancer researchers in the fields of cancer prevention on the existing gaps in cancer risk evidence in Uganda and provide direction for research priorities. This study further provided a comprehensive scope of existing cancer risk evidence and the individual and health system barriers to cancer risk reduction efforts specifically for cervical, breast, and prostate cancer prevention in Uganda. The current perspectives on priorities for cancer risk appraisal in Uganda is also recommended in this study. The limitation to this study centers on the fact that since there are limited funding opportunities for cancer research in Uganda, some of the studies that are conducted in universities and hospitals remain shelved in libraries due to limited funding support for publication. Therefore, since such studies are not archived in the online databases, this review could not access them.

## Conclusions

The unmet need for comprehensive cancer risk and prevention studies is enormous in Uganda. Future studies need to comprehensively investigate the known and putative cancer risk factors and prioritize the application of the higher-hierarchy evidence-generating epidemiological studies. Future research should prioritize comprehensive studies on etiology of the leading cause of cancer mortality, population attributable fractions, trends in cancer risk factors prevalence, the age-period-cohort effect analysis, behavior change trials, genome-wide association studies, and molecular and microbiol-pathological epidemiology using higher hierarchy of epidemiological evidence. This will guide future planning or review of a national cancer control program.

## Data Accessibility Statements

All relevant data are within the paper.
